# Artificial Intelligence-Driven Volumetric Analysis of Muscle Mass as a Predictor of Tumor Response to Neoadjuvant Chemoradiotherapy in Patients with Rectal Cancer

**DOI:** 10.3390/jcm13237018

**Published:** 2024-11-21

**Authors:** Minsung Kim, Sang Min Lee, Il Tae Son, Jaewoong Kang, Gyoung Tae Noh, Bo Young Oh

**Affiliations:** 1 Department of Surgery, Hallym Sacred Heart Hospital, Hallym University College of Medicine, Anyang 14068, Republic of Korea; bongkay4@gmail.com (M.K.); 1tae99@hanmail.net (I.T.S.); 2 Department of Radiology, CHA Gangnam Medical Center, CHA University College of Medicine, Seoul 06135, Republic of Korea; twin393@hanmail.net; 3 Medical Artificial Intelligence Center, Hallym Sacred Heart Hospital, Hallym University College of Medicine, Anyang 14068, Republic of Korea; jaewoong@mach.hallym.or.kr; 4 Department of Surgery, Ewha Womans University College of Medicine, Seoul 07804, Republic of Korea; nogang@ewha.ac.kr

**Keywords:** rectal cancer, neoadjuvant chemoradiotherapy, volumetric sarcopenia, skeletal muscle index

## Abstract

**Background/Objectives:** Artificial intelligence (AI)-based volumetric measurements for assessing sarcopenia are expected to offer comprehensive insight into three-dimensional muscle volume and distribution. Therefore, we investigated the role of sarcopenia using computed tomography (CT)-based automated AI volumetric muscle measurements in predicting neoadjuvant chemoradiotherapy (nCRT) response and prognosis in patients with rectal cancer who underwent nCRT. **Methods:** We retrospectively analyzed the data of patients who underwent nCRT followed by curative resection between March 2010 and August 2021. Sarcopenia was defined using the Q1 cutoff value of the volumetric skeletal muscle index (SMI). The association between pre-nCRT volumetric sarcopenia and nCRT response was analyzed using logistic regression. A Cox proportional hazards model was used to identify the prognostic value of the pre- and post-nCRT volumetric SMIs. **Results:** Notably, 22 (25.6%) of the 86 patients had volumetric sarcopenia. The sarcopenia group showed a poorer nCRT response than the non-sarcopenia group. Pre-nCRT sarcopenia was a significant predictor of poor nCRT response (OR, 0.34 [95% CI, 0.12–0.96]; *p* = 0.041). Furthermore, an increased volumetric SMI during nCRT was a more significant prognostic factor on recurrence-free survival (aHR, 0.26 [95% CI, 0.08–0.83]; *p* = 0.023) and overall survival (aHR, 0.41 [95% CI, 0.17–0.99]; *p* = 0.049) than a decreased SMI. **Conclusions:** Volumetric sarcopenia can be used to predict poor nCRT response. A reduction in volumetric sarcopenia can be a poor prognostic factor in patients with rectal cancer who undergo nCRT.

## 1. Introduction

Colorectal cancer is the third most common malignancy worldwide, significantly impacting morbidity and mortality [1]. Rectal cancer accounts for approximately 30% of all colorectal cancers [2]. Neoadjuvant chemoradiotherapy (nCRT) followed by surgical resection is the standard multimodal treatment [3]. This treatment strategy aims to downstage tumors, improve the feasibility of surgical resection, and reduce the risk of local recurrence [4,5,6]. Notably, numerous factors influence the prognosis of rectal cancer, and the response to nCRT is particularly significant. A good response to nCRT, defined as tumor downstaging or complete response, is known to be associated with improved prognosis, and efforts have been made to predict nCRT response [7,8]. Sarcopenia, defined as the decline in skeletal muscle mass and function, has been recognized as a significant factor associated with poorer survival outcomes, higher rates of surgical complications, and increased treatment-related toxicities in patients with rectal cancer [9,10]. Additionally, recent studies have examined the potential role of pre-nCRT sarcopenia as a predictor of treatment response in patients undergoing nCRT for rectal cancer [11,12].

Sarcopenia has traditionally been assessed using bioelectrical impedance analysis, dual-energy radiographic absorptiometry, and computed tomography (CT). Among these, CT is preferred, due to its ability to precisely quantify body composition elements, such as muscle and fat tissues [13,14]. CT scans taken at the level of the third lumbar vertebra (L3) are commonly utilized to measure skeletal muscle mass, providing a reliable method for evaluating sarcopenia in cancer patients [15]. However, there are concerns regarding the limited representation of a single slice [14]. Advancements in technology have enabled the development of automated and rapid volumetric measurements of CT scans, providing more abundant and accurate information than traditional single-slice measurements of cross-sectional images [14,16]. Artificial intelligence (AI)-based volumetric measurements for assessing sarcopenia are expected to offer comprehensive insights into three-dimensional muscle volume and distribution [17,18]. This advanced technique can calculate volumes in a few minutes, potentially improving prognostic value [14,19,20].

Therefore, we aimed to investigate the role of sarcopenia using CT-based automated AI volumetric muscle measurements in predicting nCRT response in patients with rectal cancer. We also evaluated whether this advanced volumetric measurement correlated with the prognosis of patients with rectal cancer undergoing nCRT.

## 2. Materials and Methods

### 2.1. Patients

**Figure 1:**
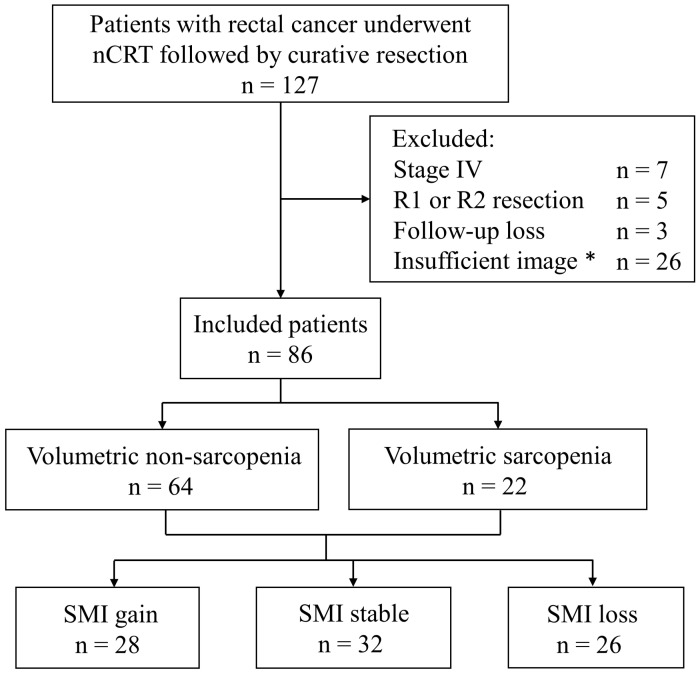
Flowchart of the study design with inclusion and exclusion criteria. * No pre-nCRT or post-nCRT CT images. nCRT = neoadjuvant chemoradiotherapy, SMI = skeletal muscle index.

Medical records of patients with rectal cancer who received nCRT followed by curative radical resection at Hallym University Sacred Heart Hospital between March 2010 and August 2021 were retrospectively reviewed. Patients diagnosed with stage IV rectal cancer, those with circumferential resection margins < 1 mm or those with positive margins, as reported in the pathological examination of surgical specimens, were excluded. We also excluded patients with incomplete CT images (missing pre- or post-nCRT CT images) or those lacking follow-up data (Figure 1). For patients diagnosed with rectal cancer located within 15 cm of the anal verge (AV) using colonoscopy, abdominopelvic CT, and pelvic magnetic resonance imaging (MRI), a multidisciplinary team decided whether to treat them with nCRT. The nCRT regimen consisted of 28 fractions of 50.4 Gy radiation combined with fluorouracil-based chemotherapy. The response to nCRT was evaluated using abdominopelvic CT and rectal MRI 4–6 weeks after completing nCRT. Patients underwent surgery 6–8 weeks after nCRT, followed by adjuvant chemotherapy, based on the pathological stage (ypStage). Clinicopathological data were collected for each patient, including age at diagnosis, sex, body mass index, preoperative carcinoembryonic antigen level, pre-nCRT clinical stage (cStage), post-nCRT clinical stage (ycStage), and ypStage, according to the American Joint Committee on Cancer 8th edition. Postoperative surveillance consisted of an abdominopelvic CT and a chest CT every six months, along with colonoscopy one year after surgery and then every two years for a total of five years. This study was approved by the Institutional Review Board of Hallym University Sacred Heart Hospital (IRB No. 2020-07-044) and was conducted in accordance with the principles of the Declaration of Helsinki. Due to its retrospective nature, the requirement for informed consent was waived by the institutional review board.

### 2.2. Assessment of Sarcopenia

**Figure 2:**
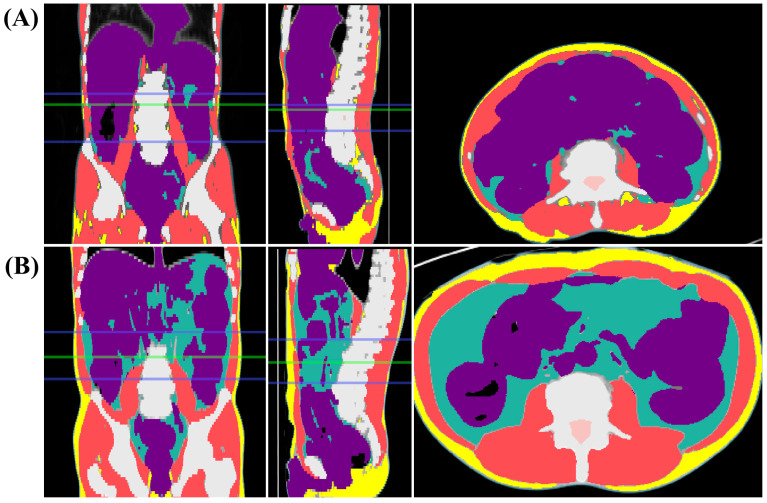
Assessment of body composition through computed tomography (CT) images. (**A**) CT images of patients classified with volumetric sarcopenia. (**B**) CT images of patients classified with volumetric non-sarcopenia. The green line marks the L3 vertebral level, while the region between the two blue lines represents the abdominal waist. CT images display skeletal muscle (red), abdominal visceral fat (green), subcutaneous fat (yellow), and visceral organs (purple) within the defined area.

This study used the same methods as our previous research [14]. An AI specialist performed automated volumetric segmentation of body composition in CT images using commercially available AI-based software for body composition analysis (DeepCatch version 1.3.0; MEDICALIP Co., Ltd., Seoul, Republic of Korea). This demonstrated a 97% accuracy rate, compared with manual segmentation, the gold standard [18]. The software employed a three-dimensional U-NET [21,22] model for segmentation, which was trained using semiautomatic segmentation data of muscle, abdominal visceral fat, and subcutaneous fat. For skeletal muscle, Hounsfield unit (HU) thresholds of −29–150 were applied. Anonymized pre-contrast abdominopelvic CT images were uploaded to the software. The software automatically identified the abdominal waist and provided color-coded maps of body composition, including skeletal muscle, abdominal visceral fat, and subcutaneous fat (Figure 2). According to World Health Organization guidelines, the abdominal waist is defined as the area between the lower edge of the thoracic ribs and the upper boundary of the iliac crest [23]. A radiologist reviewed and adjusted the automatic segmentation and localization results for the abdominal waist to ensure accuracy. The software quantified the volume (cm^3^) of the skeletal muscles in the abdominal waist region. The volumetric skeletal muscle index (SMI) was calculated by dividing the waist muscle volume (cm^3^) by the cubic value of height (m^3^) [16,20]. There are limited studies on volumetric parameters; therefore, the optimal cutoff values for the SMI in diagnosing sarcopenia were determined using the first quartile (Q1) values from a reference study [14,16]. The Q1, representing the 25th percentile of the sample, was used as the cutoff value for muscle mass in the absence of established optimal cutoffs [24,25,26]. Patients with Q1 values were classified as having sarcopenia, whereas others were classified as having non-sarcopenia. We also evaluated the magnitude of change in the volumetric SMI before and after nCRT. Patients with a >5% increase or reduction in the volumetric SMI were classified as the gain and loss groups, respectively. The remaining patients were classified as the stable group [27].

### 2.3. Assessment of Clinical Outcome

Three gastrointestinal radiologists with >15 years of experience evaluated the clinical tumor response to nCRT by comparing the MRI results before and after nCRT. T or N downstaging was defined as a good response, and no change or worsening of the tumor was defined as a poor response. We analyzed the relationship between the pre-nCRT volumetric SMI (sarcopenia vs. non-sarcopenia) and nCRT response (good vs. poor responders). We also analyzed survival outcomes regarding recurrence-free survival (RFS) and overall survival (OS) according to the volumetric SMI (sarcopenia vs. non-sarcopenia) and volumetric SMI change types (gain vs. stable vs. loss). The primary endpoint was the predictive value of the pre-nCRT volumetric SMI for the nCRT response, and the secondary endpoint was the prognostic value of the pre- and post-nCRT volumetric SMIs.

### 2.4. Statistical Analysis

The baseline characteristics and survival outcomes of the sarcopenia and non-sarcopenia groups were analyzed. Fisher’s exact test was used for categorical variables, whereas Student’s *t*-test was used for continuous variables. Univariate and multivariate logistic regression analyses of nCRT responses were used to calculate odds ratios (OR) and 95% confidence intervals (CIs). Kaplan–Meier survival curves for RFS and OS were plotted for the SMI groups and SMI change types and compared using the log-rank test. Univariate and multivariate analyses of survival outcomes were performed using the Cox proportional hazards model to calculate the adjusted hazard ratios (aHR) and 95% CIs. Statistical significance was set at a *p*-value of <0.05. All statistical analyses were performed using the SPSS software (version 25.0; SPSS Inc., Chicago, IL, USA).

## 3. Results

### 3.1. Patient Characteristics

Overall, 86 patients were included in the study. According to the Q1 cutoff value for the volumetric SMI (sarcopenia: SMI < 154.65 cm^3^/m^3^ and <184.19 cm^3^/m^3^ in males and females, respectively), the non-sarcopenia and sarcopenia groups included 64 (74.4%) and 22 (25.6%) patients, respectively. Table 1 shows the patients’ clinicopathological characteristics. The patients in the sarcopenia group were older than those in the non-sarcopenia group (69.9 ± 11.3 vs. 61.8 ± 10.7, *p* = 0.004), and the sex distribution did not differ between the two groups. Additionally, the cStage and ypStage did not differ between the two groups.

### 3.2. Pre-nCRT Volumetric SMI and nCRT Response

We analyzed the tumor response according to the volumetric pre-nCRT SMI to investigate the relationship between volumetric sarcopenia and nCRT response. The non-sarcopenia group exhibited a higher rate of good responders than the sarcopenia group (70.3% vs. 29.7%, *p* = 0.043) (Table 1). Table 2 presents the factors associated with nCRT response, based on logistic regression analysis. The sarcopenia group had a poorer response (OR, 0.34 [95% CI, 0.12–0.96]; *p* = 0.041) compared with the non-sarcopenia group. These findings suggest that the pre-nCRT volumetric SMI significantly predicts response to nCRT in patients with rectal cancer.

### 3.3. Volumetric SMI Change and Survival Outcomes

**Figure 3:**
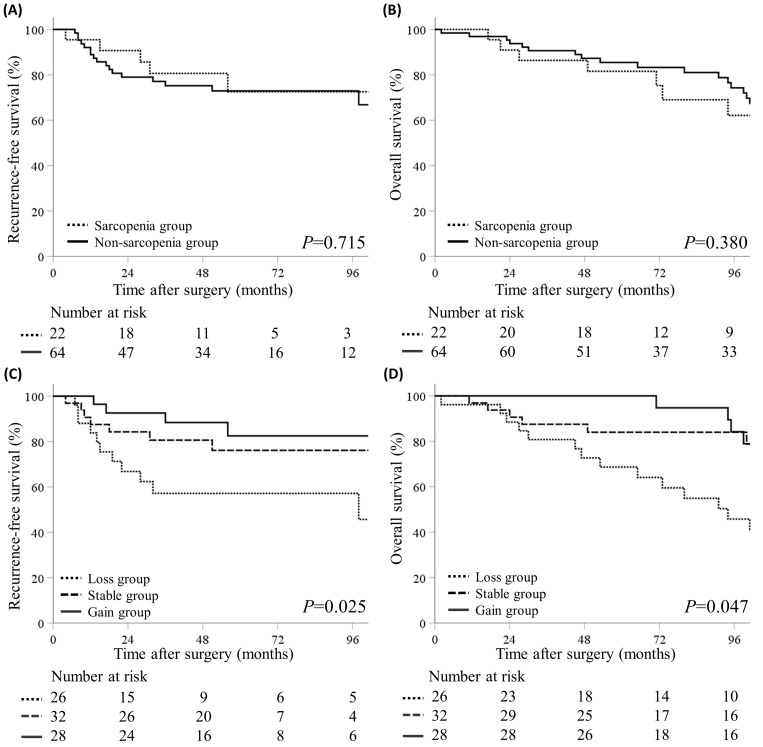
Kaplan–Meier curves for recurrence-free survival (RFS) and overall survival (OS). (**A**) RFS based on the pre-nCRT volumetric skeletal muscle index (SMI). (**B**) OS according to the pre-nCRT volumetric SMI. (**C**) RFS according to volumetric SMI change types (increase or reduction in the SMI of over 5%). (**D**) OS based on volumetric SMI change types. *p*-values are from the log-rank test.

Survival analysis was performed to identify the prognostic value of the volumetric SMI. The median follow-up duration was 48.5 months. We analyzed the RFS and OS between the pre-nCRT sarcopenia and non-sarcopenia groups and found no significant differences (Figure 3A,B). We also analyzed the relationship between changes in the SMI and survival. The patients were categorized into three groups based on a volumetric SMI change > 5% before and after nCRT: loss (n = 26), stable (n = 32), and gain groups (n = 28) (Figure 1). The RFS significantly differed among the three groups, with the worst and best survival in the loss and gain groups, respectively (*p* = 0.025) (Figure 3C). Similarly, the OS significantly differed among the three groups (*p* = 0.047) (Figure 3D).

Table 3 shows the factors associated with survival outcomes, based on the Cox proportional hazards model. The gain group had a better RFS (aHR, 0.26 [95% CI, 0.08–0.83]; *p* = 0.023) and OS (aHR, 0.41 [95% CI, 0.17–0.99]; *p* = 0.049), compared with the loss group. However, no significant difference was observed in the pre-nCRT SMI between the sarcopenia and non-sarcopenia groups. These findings suggest that the SMI change before and after nCRT is a significant prognostic factor for survival, but the pre-nCRT SMI is not.

## 4. Discussion

In this study, we investigated using AI-based volumetric measurements to assess sarcopenia and its impact on patients with rectal cancer who underwent nCRT. The pre-nCRT volumetric SMI was a significant predictor of nCRT response, and the SMI change during nCRT was a significant prognostic factor for survival. An AI-based volumetric muscle measurement offers more precise and comprehensive insights than a traditional L3 cross-sectional single-slice measurement and is crucial for predicting nCRT response and survival outcomes. This advanced technique highlights the importance of providing accurate prognostic information for the treatment of rectal cancer.

Sarcopenia is consistently associated with poor survival outcomes in patients with cancer [9,10,28]. However, there is no accepted optimal cutoff value for defining sarcopenia, leading to the use of various thresholds in different studies. A commonly used threshold is the Martin cutoff value at the L3 vertebral level [15,29,30]. However, other studies have applied the Q1 cutoff value at the same level [24,25]. Takeda et al. [31] reported that pre-nCRT sarcopenia, according to the Q1 cutoff value at the L3 vertebral level, was an independent negative prognostic factor in patients with rectal cancer who underwent nCRT. Recent studies have attempted volumetric measurements and applied the Q1 cutoff value to define sarcopenia [14,16,20].

Furthermore, the volumetric SMI may provide a more accurate insight into sarcopenia than the L3 SMI [20,26]. Single-slice image analysis has limitations due to constant shifts in the contents of the gastrointestinal tract, making it difficult to capture consistent anatomy in repeated L3 sections. Additionally, muscle area measurements can vary significantly at different abdominal levels, sometimes doubling the actual value. Therefore, volumetric measurements offer greater precision than single-slice measurements at the L3 level [16,19]. The cutoff values for sarcopenia vary for several reasons. One is the reference population, as body sizes may differ significantly between Eastern and Western populations. These variations can lead to different cutoff values, resulting in different prevalence rates of sarcopenia and, thus, different results [32]. In this study, the Q1 cutoff value was employed for volumetric sarcopenia to investigate its association with nCRT response. Furthermore, several studies have reported an association between changes in the SMI during surgery or nCRT and the prognosis of patients with rectal cancer [27,33,34,35]. These studies employed different methods of change in muscle mass during treatment. De Nardi et al. [34] established 2% and 5% variation thresholds, and Fukuoka et al. [35] and Zhang et al. [27] employed a 10% variation threshold. Chung et al. [2] employed a −4.2% decrease. However, in this study, we divided patients into three groups based on a 5% change in the volumetric SMI to evaluate its correlation with survival outcomes.

A good response to nCRT is associated with a better prognosis in patients with rectal cancer [7,8]. In particular, achieving a complete response to nCRT can allow for considering a “watch and wait” strategy, which has recently been increasingly explored as a treatment option [3,36]. This has led to numerous studies investigating factors associated with nCRT response, including the relationship between the SMI and nCRT response. A previous study reported that sarcopenia does not predict poor nCRT response [11]. However, another study reported that pre-nCRT sarcopenia was an independent predictor of poor total neoadjuvant therapy in patients with rectal cancer [12]. In this study, pre-nCRT volumetric sarcopenia was an independent predictive factor of poor nCRT response. Unlike previous studies, the present study did not find a significant association between pre-nCRT volumetric sarcopenia and survival. This lack of association between pre-nCRT sarcopenia and survival outcomes, despite its relationship with nCRT response, may be attributed to the relatively small sample size of our study, which could limit the statistical power to detect significant correlations. Additionally, confounding factors such as comorbidities, nutritional status, and postoperative complications might have influenced survival outcomes independently. Furthermore, the observed significant association between tumor response and survival outcomes in our study population (*p* = 0.012 for RFS and *p* = 0.027 for OS) suggests that tumor response plays a critical role in prognosis. However, the indirect relationship between sarcopenia, tumor response, and survival outcomes requires further investigation to clarify the underlying mechanisms. We observed that the degree of change in muscle volume during nCRT, rather than pre-nCRT sarcopenia, significantly affected survival. Previous studies have reported the prognostic significance of the SMI during surgery or nCRT in patients with rectal cancer [2,27,33,34,35].

From a biological perspective, sarcopenia may indicate increased metabolic activity in more aggressive tumors, leading to systemic inflammation and subsequent muscle loss. This change in body composition may reflect a distinct biological response to cancer therapy, suggesting that effective chemotherapy could potentially reverse the catabolic processes responsible for cachexia. Conversely, a significant reduction in skeletal muscle mass during treatment might indicate a more aggressive form of the disease and the possible ineffectiveness of neoadjuvant therapy [32]. Consistent with these findings, our study found that changes in the volumetric SMI during nCRT were associated with survival outcomes. Particularly, this study reported that patients who experienced an increase in the volumetric SMI during nCRT had better RFS and OS rates than those with a decreased volumetric SMI. This finding suggests the importance of monitoring and possible interventions in muscle mass changes during treatment to improve the survival outcomes of the patients. Therefore, early recognition and intervention may help treat patients with colorectal cancer [37]. Furthermore, prehabilitation becomes essential, including preoperative interventions focused on physical activity, nutrition, and psychosocial support. These strategies are designed to mitigate muscle loss caused by the catabolic effects of cancer, leading to reduced complications and enhanced quality of life for patients [38].

This study has some limitations. First, this was a retrospective study; therefore, it was subject to selection bias and may not accurately represent the general population. Additionally, potential confounding factors, such as nutritional status and comorbidities, were not comprehensively controlled, due to the lack of detailed and consistent data. Second, the sample size was relatively small, with only 86 patients from a single institution, which may limit the generalizability of the findings and the statistical power to detect significant differences. As such, this study emphasizes the need for larger, multicenter studies to validate these findings and ensure broader applicability. Based on these results, we are planning a multicenter study to further investigate the role of volumetric sarcopenia in rectal cancer treatment. Third, we could not elucidate the mechanisms underlying the observed association between volumetric sarcopenia, nCRT response, and survival outcomes. Therefore, further prospective studies with larger sample sizes, more comprehensive data collection, and a focus on biological mechanisms are needed to validate our findings and provide more comprehensive insights into the role of sarcopenia in rectal cancer treatment and prognosis. To our knowledge, this is the first study to examine the prognostic significance of volumetric sarcopenia in patients with rectal cancer who have undergone nCRT. Volumetric measurements provided more detailed and precise information than single-slice measurements, and the evaluation was simplified by automated calculations. While this study highlights the potential advantages of volumetric measurement, a direct comparison between volumetric and single-slice techniques is necessary to validate the clinical superiority and predictive accuracy of volumetric analysis. Future research should focus on conducting such comparative studies to further elucidate the benefits and limitations of each method, ensuring their optimal application in clinical practice.

In conclusion, in the present study, we found that pre-nCRT volumetric sarcopenia, automatically assessed using AI-based software, was associated with a poor nCRT response. A reduction in the volumetric SMI during nCRT resulted in a worse RFS and OS than an increase in the volumetric SMI. These volumetric muscle mass measurements can be easily obtained, and physicians should pay attention to patients with poor prognostic features.

## Figures and Tables

**Table 1:** Characteristics of the patients.

Variables	Sarcopenia (n = 22)	Non-Sarcopenia (n = 64)	*p*-Value
Age	69.9 ± 11.3	61.8 ± 10.7	0.004
Sex			1
Male	15 (68.2)	44 (68.8)	
Female	7 (31.8)	20 (31.2)	
BMI (kg/m^2^)	21.8 ± 2.3	24.2 ± 3.2	0.003
cStage			1
I	1 (4.5)	4 (6.3)	
II	6 (27.3)	17 (26.6)	
III	15 (68.2)	43 (67.2)	
CEA (ng/mL)			1
≥5.0	4 (18.2)	13 (19.1)	
<5.0	18 (81.8)	55 (80.9)	
ypStage			0.863
0 or I	8 (36.4)	24 (37.5)	
II	9 (40.9)	22 (34.4)	
III	5 (22.7)	18 (28.1)	
Cell type			1
WD/MD	21 (95.5)	59 (92.2)	
PD/MUC/SRC	1 (4.5)	5 (7.8)	
LVI			1
Positive	5 (22.7)	16 (25.0)	
Negative	17 (77.3)	48 (75.0)	
PNI			1
Positive	2 (9.1)	6 (9.4)	
Negative	20 (90.9)	58 (90.6)	
nCRT response			0.043
Poor	12 (54.5)	19 (29.7)	
Good	10 (45.5)	45 (70.3)	

BMI, body mass index; cStage, clinical stage before chemoradiotherapy; CEA, carcinoembryonic antigen; ypStage, pathologic stage after chemoradiotherapy from a surgical specimen; WD, well differentiated; MD, moderately differentiated; PD, poorly differentiated; MUC, mucinous; SRC, signet ring cell; LVI, lymphovascular invasion; PNI, perineural invasion; nCRT, neoadjuvant chemoradiotherapy.

**Table 2:** Logistic regression analyses of the predictive factors for good response to nCRT.

Parameter	Univariate		Multivariate	
	OR (95% CI)	*p*-Value	OR (95% CI)	*p*-Value
Pre-nCRT SMI				
Non-sarcopenia	1 (Reference)		1 (Reference)	
Sarcopenia	0.35 (0.13–0.95)	0.04	0.34 (0.12–0.96)	0.041
Age				
<65	1 (Reference)		1 (Reference)	
≥65	1.14 (0.46–2.80)	0.778	2.10 (0.94–4.69)	0.072
Sex				
Male	1 (Reference)		1 (Reference)	
Female	1.52 (0.57–4.03)	0.403	2.44 (0.99–6.00)	0.053
cStage		0.066		
I	1 (Reference)			
II	1.09 (0.48–2.47)	0.835		
III	1.90 (1.11–3.27)	0.02		
CEA				
<5.0	1 (Reference)			
≥5.0	0.82 (0.26–2.55)	0.726		

OR, odds ratio; CI, confidence interval; SMI, skeletal muscle index; nCRT, neoadjuvant chemoradiotherapy; cStage, clinical stage before chemoradiotherapy; CEA, carcinoembryonic antigen.

**Table 3:** Cox proportional hazard analyses of the prognostic factors for survival outcomes.

Parameter	Recurrence-Free Survival				Overall Survival			
	Univariate		Multivariate		Univariate		Multivariate	
	cHR (95% CI)	*p*-Value	aHR (95% CI)	*p*-Value	cHR (95% CI)	*p*-Value	aHR (95% CI)	*p*-Value
SMI change		0.035		0.035		0.063		0.07
Loss group	1 (Reference)		1 (Reference)		1 (Reference)		1 (Reference)	
Stable group	0.43 (0.17–1.11)	0.081	0.38 (0.15–1.01)	0.051	0.38 (0.15–0.94)	0.037	0.41 (0.17–1.03)	0.058
Gain group	0.25 (0.8–0.80)	0.019	0.26 (0.08–0.83)	0.023	0.44 (0.19–1.06)	0.068	0.41 (0.17–0.99)	0.049
Pre-nCRT SMI								
Non-sarcopenia	1 (Reference)				1 (Reference)			
Sarcopenia	0.83 (0.31–2.25)	0.716			1.42 (0.65–3.13)	0.382		
Age								
<65	1 (Reference)				1 (Reference)		1 (Reference)	
≥65	1.53 (0.66–3.53)	0.321			2.34 (1.11–4.94)	0.026	2.75 (1.26–5.99)	0.011
Sex								
Male	1 (Reference)				1 (Reference)			
Female	0.61 (0.23–1.67)	0.337			0.66 (0.28–1.54)	0.334		
cStage		0.633				0.59		
I	1 (Reference)				1 (Reference)			
II	1.36 (0.16–11.72)	0.779			2.72 (0.35–21.12)	0.338		
III	2.01 (0.26–15.26)	0.501			2.18 (0.29–16.49)	0.451		
CEA (ng/mL)								
<5.0	1 (Reference)		1 (Reference)		1 (Reference)			
≥5.0	2.19 (0.89–5.39)	0.087	2.39 (0.93–6.15)	0.071	1.25 (0.47–3.31)	0.653		
ypStage		0.064		0.058		0.773		
0 or I	1 (Reference)		1 (Reference)		1 (Reference)			
II	2.36 (0.73–7.70)	0.153	3.17 (0.93–10.83)	0.066	1.32 (0.56–3.14)	0.529		
III	3.76 (1.16–12.22)	0.028	3.49 (1.07–11.40)	0.038	1.36 (0.52–3.55)	0.536		
Cell type								
WD/MD	1 (Reference)				1 (Reference)			
PD/MUC/SRC	1.50 (0.35–6.43)	0.584			1.67 (0.50–5.55)	0.404		
LVI								
Negative	1 (Reference)				1 (Reference)		1 (Reference)	
Positive	2.04 (0.85–4.86)	0.109			2.11 (0.95–4.70)	0.067	2.90 (1.24–6.80)	0.014
PNI								
Negative	1 (Reference)				1 (Reference)			
Positive	3.29 (1.10–9.82)	0.033			2.63 (0.77–8.99)	0.123		

cHR, crude hazard ratio; aHR, adjusted hazard ratio; CI, confidence interval; SMI, skeletal muscle index; nCRT, neoadjuvant chemoradiotherapy; cStage, clinical stage before chemoradiotherapy; CEA, carcinoembryonic antigen; ypStage, pathologic stage after chemoradiotherapy from the surgical specimen; WD, well differentiated; MD, moderately differentiated; PD, poorly differentiated; MUC, mucinous; SRC, signet ring cell; LVI, lymphovascular invasion; PNI, perineural invasion.

## Data Availability

The datasets generated or analyzed during the study are available from the corresponding author upon reasonable request.
